# Supplementary material for: Polygenic Risk Score in Predicting Esophageal, Oropharyngeal, and Hypopharynx Cancer Risk among Taiwanese Population
Source: Cancers (Basel). 2024 Feb 7;16(4):707. doi: 10.3390/cancers16040707 (PMC10886704; doi:10.3390/cancers16040707)
Supplement: Supplementary file 1 [file cancers-16-00707-s001.zip › cancers-2774583-supplementary.pdf]

Supplementary Figure S1. Distribution of PGS001087 (A) and PGS001394 (B) by incident SCC.

(A) PGS0001087 (B) PGS0001394

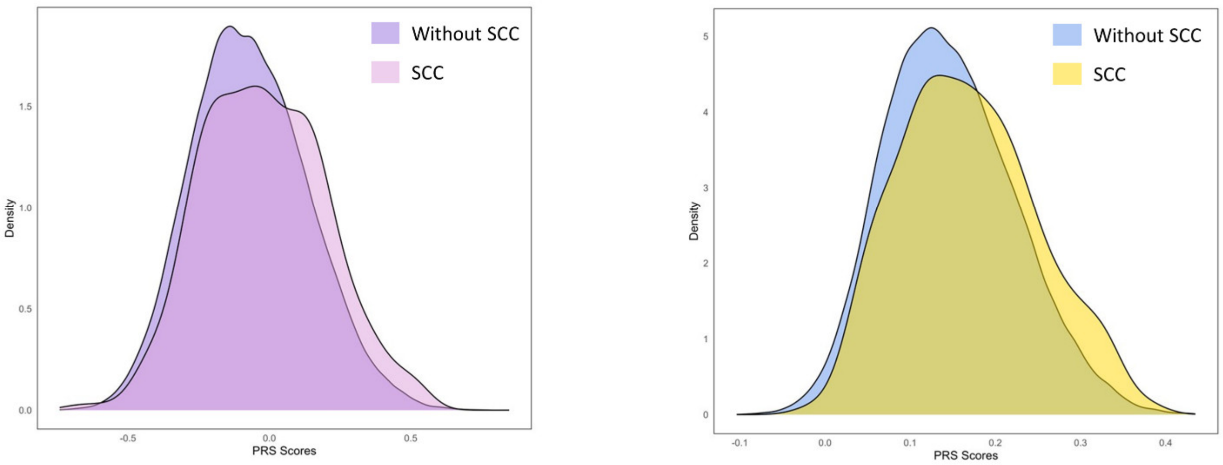

Supplementary Table S1. Risk of various polygenic risk scores for first-onset SCC.

Polygenic Risk Scores (PRS)	Reported Trait	HR[a]	95% CI		*p* value
PGS002063	Esophagitis, GERD and related diseases	1.120	0.002	6.844	0.973
PGS001046	Past tobacco smoking (Smoked at least once)	1.563	0.895	2.730	0.117
PGS001047	Past tobacco smoking (Smoked occasionally)	1.132	0.506	2.532	0.762

[a] HRs were estimated using Cox proportional hazards model adjusted for age and gender.

Supplementary Table S2. Classification of the study population with the three squamous cell carcinomas (SCC) by quartile with PGS001394.

Variables (SCC)		Quartiles of the polygenic risk score (PGS001394)								*p* value[a]
		Q1 (n = 13384)		Q2 (n =13742)		Q3 (n = 13571)		Q4 (n = 13265)		
		N	%	N	%	N	%	N	%	
Esophagus										<0.001
	NO	13361	99.83	13697	99.7	14527	99.7	13186	99.4	
	Yes	23	0.17	45	0.33	44	0.30	79	0.60	
Oropharynx										0.638
	NO	13357	99.80	13708	99.75	14534	99.75	13228	99.72	
	Yes	27	0.20	34	0.25	37	0.25	37	0.28	
Hypopharynx										0.171
	NO	13366	99.87	13714	99.80	14541	99.79	13231	99.74	
	Yes	18	0.13	28	0.20	30	0.21	34	0.26	

[a] Categorical variables were expressed as numbers (percent) and were analyzed using the Chi-square test.
